# Co-design of Digital Health Interventions for Young Adults: Protocol for a Scoping Review

**DOI:** 10.2196/38635

**Published:** 2022-10-24

**Authors:** Jessica A Malloy, Stephanie R Partridge, Joya A Kemper, Andrea Braakhuis, Rajshri Roy

**Affiliations:** 1 Discipline of Nutrition & Dietetics Faculty of Medical & Health Sciences University of Auckland Auckland New Zealand; 2 Westmead Applied Research Centre Faculty of Medicine and Health The University of Sydney Sydney Australia; 3 Prevention Research Collaboration Sydney School of Public Health The University of Sydney Sydney Australia; 4 Department of Management, Marketing, and Entrepreneurship University of Canterbury Business School University of Canterbury Christchurch New Zealand

**Keywords:** digital health intervention, eHealth, mHealth, digital intervention, co-design, user participation, participatory research, participatory medicine, user feedback, participatory design, social media, web-based tool, young adult, youth, teenager, adolescent, review, protocol, search strategy, medical librarian, health librarian, library science, information science

## Abstract

**Background:**

Digital health interventions, including apps and web-based services, are on the rise due to their facilitated access to target groups. The constant evolution of technology calls for participatory research methodologies to understand youth expectations and the use of technology. The creative and collaborative nature of co-design allows for the active integration of youth desires and may enhance acceptability when it comes to digital health tools.

**Objective:**

The primary objective of this review is to assess the breadth of literature on digital health interventions that have been co-designed for and by young adults, including the types of available evidence, the identification of key characteristics relevant to young adult co-design, and the examination of research conduct in this space.

**Methods:**

The proposed scoping review will be conducted in accordance with the Joanna Briggs Institute (JBI) Manual for Scoping Reviews. As well as the PRISMA-ScR (Preferred Reporting Items for Systematic Reviews and Meta-Analyses Extension for Scoping Reviews) checklist for reporting scoping reviews, an adaptation of Arksey and O’Malley’s 6-stage framework for scoping reviews will be referenced. Peer-reviewed primary research, where young adults (aged 15-35 years) were actively involved in the design and development process of digital health interventions, will be collated for analyses. Five databases, including MEDLINE (Ovid), Cochrane, CINAHL Plus, Google Scholar, and Scopus, will be searched for relevant papers. Search strategies will be comprehensive to identify both published and unpublished literature. Relevant gray literature and secondary research will be excluded but pooled for separate analysis and citation chaining. Results will be presented in one or multiple forms, including narrative, tabular, or diagrammatic.

**Results:**

Data collection commenced in October 2021. Following data extraction according to the JBI results extraction instrument and independent quality assurance of included studies, a narrative synthesis of each paper included in the final pool will allow for data charting. As of May 2022, 19 papers are included for analysis. We expect the results to be published by autumn 2022.

**Conclusions:**

This protocol provides guidance for researchers who plan to conduct a similar style of investigation and promotes standardization of the scoping review process. We anticipate the provision of an overview of participatory digital health research involving young adults, highlighting any gaps in this research area, as well as potential areas for further study.

**International Registered Report Identifier (IRRID):**

DERR1-10.2196/38635

## Introduction

### Background and Rationale

Digital technologies present ever-evolving opportunities for health professionals and researchers to effectively engage with young adults (YAs) online. Regardless of socioeconomic status, the vast majority of young people have access to a mobile device, with which they spend up to 10 hours per day interacting [1,2]. As such, smartphones and their capabilities, as well as personal computers with internet access, provide promising and potentially equitable platforms to influence a vast range of health behaviors.

Young adulthood is a critical development period, one in which health ethos and behaviors are, often for the first time, shaped outside of the home [3]. Health habits and behaviors that are adopted during this period often have a profound impact on long-term health status and general well-being [4].

Recent studies that have explored the health-seeking behaviors of YAs state a reliance on the internet for health information [5-7]. Actors in this space, including health influencers as well as health practitioners, are increasingly trusted with the provision of health information online [7]. The evolution of social media continues to add a more interactive element, as well as multiplying opportunities to encounter misinformation or succumb to “echo chambers” or confirmation bias [8]. The presence of health professionals in the digital space, via intervention apps or websites, introduces an opportunity to influence health behaviors online while also allowing for the dissemination of evidence-based health information in an environment where the voices of health professionals are typically outnumbered [9]. In this review, “digital technology” is used as an umbrella term that encompasses smartphone apps, SMS text messaging, social media, and web use, or a combination of multiple mediums.

Co-design, which originates from participatory research, has been defined as a “process of collective creativity or partnership with potential users and stakeholders, who are actively involved in the development of the technology, helping to ensure it meets the users’ needs and preferences” [10]*.* Co-design with future users (FUs) enables researchers and designers to determine the attitudes and values of FUs, as well as their interests and capabilities [11]. An important distinction from a user-centered approach, co-design not only develops interventions *for* end users but also designs *with* users, consulting with target groups throughout the development process. The more creative and collaborative nature of co-design enables “greater participation and more effective responses to complex health issues” [12]. Increasingly, research uses co-design frameworks to create digital health interventions, especially for young people (ie, Young and Well Cooperative Research Centre) [13]. Where the evolution of technology is constant, participatory design methodologies are ever more valuable in the development of digital tools to sustain knowledge of youth expectations and use.

Evidence supports the notion that co-design or participatory design may enhance desired outcomes through increasing engagement and maintaining participation, while increasing the likelihood of developing a universally acceptable tool [11,14]. By way of example, Davis et al [15] coupled a patient-driven participatory design approach with professional respiratory expertise to develop a self-management app for young people with asthma. The digital app, which was found to be highly acceptable to young co-designers, highlights how co-design may result in more engaging, accessible, and acceptable intervention tools.

Prior reviews have explored the employment of co-design methodologies in the creation of digital health interventions for children and adolescents [10,16]. Further, a recent scoping review protocol seeks to investigate strategies for adolescent engagement in digital health interventions for obesity management [17]. Researchers have called for more participatory research in the development of such interventions due to an observed influence on engagement and subsequent health behaviors [16]. While research on the co-design of digital health interventions is gaining momentum, particularly for children and adolescents, there appears to be limited research concerning the young adult population. We, therefore, intend to explore the breadth of literature in this area to summarize and provide guidance for future participatory research with this age group.

### Objectives

A preliminary search of MEDLINE, the Cochrane Database of Systematic Reviews, and the Joanna Briggs Institute (JBI) Evidence Synthesis journal was conducted, and no current or underway systematic or scoping reviews on the topic were identified. Presently, evidence surrounding the YA co-design of digital health interventions is scattered across health fields. An initial search outlined that the nature of the evidence is indicative of the need for a scoping review (ScR), rather than a systematic review, to synthesize relevant research. Where systematic reviews pose precise questions addressed to an established volume of literature, scoping reviews are suited to an “emerging” evidence base. The principal objective of an ScR is to “scope” a body of literature in order to “identify knowledge gaps, clarify concepts, or investigate research conduct” [18]. The overarching objective of this ScR is to assess the breadth of literature on digital health interventions that have been co-designed for and by YAs, including the types of available evidence, identification of key characteristics relevant to YA co-design, and examination of research conducted in this space [18,19].

## Methods

### Protocol Design

The proposed ScR will be conducted in accordance with the JBI Manual for Scoping Reviews [20]. The PRISMA-ScR (Preferred Reporting Items for Systematic Reviews and Meta-Analyses Extension for Scoping Reviews) checklist will be used (Multimedia Appendix 1) [21]. The PRISMA-ScR was developed to provide guidance on the reporting of scoping reviews. This reporting guideline is consistent with the JBI guidance for scoping reviews, which highlights the importance of methodological rigor in the conduct of such reviews [20]. As well as the PRISMA-ScR checklist, an adaptation of Arksey and O’Malley’s 6-stage framework for ScR has been referenced [19,21]. The framework, which Levac et al [22] and Kahlil et al [23] adapted, consists of 5 core stages that will be used to address the primary research questions. The 5 stages include:

1. Identification of the research question
2. Identification of relevant studies
3. Selection of studies (search strategy)
4. Charting of data
5. Summary and report of research findings

### Identification of the Research Question

As aforementioned, recent reviews have contributed to the evidence base concerning co-designed digital health interventions. However, such research primarily focuses on adults or adolescents and children, with limited research in the YA space. An ScR is performed in order to collate research findings from “scattered” evidence where research is insufficient to warrant a systematic review [18]. Following the preliminary literature search, it was deemed that a systematic review would limit findings, given the reductionist approach required. As such, this ScR intends to assess the breadth of literature on youth co-design of digital health interventions in order to inform future research in the digital health space.

The PCC (population/participants, concept, context) framework is recommended to identify the main concepts of primary review questions and inform search strategy development [19].

To comprehensively investigate how co-design with YAs is used in the development of digital health interventions, the overarching objective of this review has been translated into 4 key research questions according to the PCC framework [24];

1. Which theories and frameworks of co-design or participatory design have been used to involve YAs in the development of digital health interventions?
2. What health outcomes (if available) have been improved using co-design of digital health interventions in YAs?
3. How does co-design or participatory design support behavior change efforts such as reach, engagement, and accessibility of digital health interventions for YAs?
4. What is the potential to build credibility, trust, collaboration, and advocacy online?

The nature of reporting is such that papers are typically separated by a focus on either co-design processes or health outcomes. Where possible, all relevant papers from a study will be sought in order to comprehensively assess the co-design methodology as well as associated intervention outcomes. The intent of this ScR is to collate learnings and determinants of success in the co-design of digital interventions that endeavor to improve the health of YAs. Resolving the above research questions will enable the fulfillment of the primary research objective.

### Identification of Relevant Studies

The following steps will be followed in order to obtain the final pool of studies for analysis:

1. An initial limited search of a selection of relevant databases
2. Analysis of text words contained in the title, abstract, and the keywords used to describe relevant articles
3. A second comprehensive search using all identified keywords and index terms across selected databases
4. Identification of additional studies via reference lists of all relevant reports and articles identified (citation chaining)

Search strategies have been developed via consultations with a University of Auckland science librarian (Textbox 1). Searches will be run on each selected database (n=5), as well as manual searching of gray literature to attain the final pool. Databases searched will include MEDLINE (Ovid), Scopus, CINAHL Plus, Cochrane, and Google Scholar (to be used for both an advanced and gray literature search).

**Search strategy (Cochrane) for the identification of papers for analysis.**

1. MeSH descriptor: [Community-Based Participatory Research] this term only
2. MeSH descriptor: [Community Participation] this term only
3. (Co NEXT (design* or creat* or produc* OR youth led or youth?led or participatory design* or user?centered or user?centered):ti,ab,kw (Word variations have been searched for)
4. MeSH descriptor: [Digital Technology] this term only
5. MeSH descriptor: [Social Media] this term only
6. MeSH descriptor: [Online Social Networking] this term only
7. MeSH descriptor: [Internet-Based Intervention] this term only
8. MeSH descriptor: [Internet] this term only
9. MeSH descriptor: [Blogging] in all MeSH products
10. (digital* or web?based or social media or Instagram or Facebook or Twitter or Snapchat or social app* or online or internet):ti,ab,kw (Word variations have been searched)
11. MeSH descriptor: [Young Adult] this term only
12. (young adult* or YA or youth* or young person*):ti,ab,kw (Word variations have been searched
13. 1 or 2 or 3
14. 4 or 5 or 6 or 7 or 8 or 9 or 10
15. 11 or 12
16. 13 and 14 and 15 in Cochrane Reviews, Trials with ‘Public Health’ in Cochrane Groups (Word variations have been searched)

Date of most recent search: September 2021

### Selection of Studies for Inclusion

The papers sourced will be exported to the reference management software EndNote X9 (Clarivate). Collated papers will be scanned and reviewed according to the inclusion criteria. Such criteria will be refined iteratively as the independent review is undertaken. Articles will be screened for duplicates, prior to title screening, by the primary author (JM). The remaining papers will be screened by 2 independent reviewers (JM and RR). Relevant secondary evidence will be citation chained by the primary author, with the paper source added to libraries to undergo a secondary independent abstract review. Following consultation and resolution of library discrepancies, a final list of papers eligible for full-text screening will be created. The full-text of the papers will be screened by the primary author (JM). Full-text articles will then be citation chained, with the above process repeated for newly sourced articles. The final list for inclusion will be assessed independently by 3 reviewers to ensure the eligibility criteria are upheld. Discrepancies regarding the final list will be discussed and resolved in meetings. The eligibility criteria will be iteratively refined where required, with ineligible papers excluded. The results of the search and the study inclusion process will be reported in full in a PRISMA-ScR flow diagram.

### Eligibility Criteria (PCC)

#### Types of Participants (Population)

The classification of YAs differs across the literature, with the United Nations defining the age range as 15-24 years, whereas the World Health Organization describes this age group as “youth” [25]. Initially, the population group of interest for this review was further narrowed to focus on those in “young adulthood” aged 18-24 years, a scope which is inclusive of individuals classed as either Generation Z or “young” Millennials, as defined by the Massachusetts Institute of Technology [26]. Following consultation with the review team during study selection, the age range for inclusion was iteratively widened to include participants aged 15-35 years. This iteration was applied to ensure relevant studies were not excluded. 

#### Concept

The literature will be searched for studies reporting on the participation or engagement of YAs in the design and development of interventions to improve health using digital tools or technologies. To be eligible, studies must include 2 core concepts: the use of co-design or similar methodologies, and digital health tools. For this review, “health interventions” encompass those aiming to improve chronic disease states, mental health conditions, dietary or physical activity habits, and risky health behaviors including binge drinking and smoking.

Health intervention studies that explicitly state co-design or participatory research design where there is active involvement of YAs for the duration of the research process in the form of leadership roles, design and knowledge sharing focus groups or workshops, ongoing interviews, and iterative development of the intervention will be included.

Health intervention studies that do not explicitly state co-design yet report the use of co-design or similar methodologies where the above criteria are met regarding active involvement of YAs in design and development processes will also be included for analyses. Eligible digital health interventions or technologies will include eHealth and mobile health (mHealth), and those that are web-based or include social networking, blogging, engagement with social media, SMS text messaging, or general digital health communications.

#### Context

Peer-reviewed empirical research in all languages will be included and translated if required. Literature prior to 2006 will be excluded due to the emergence of social media and modern apps, and to ensure relevancy to YAs.

#### Types and Sources of Literature

Published peer-reviewed primary research (including both qualitative and quantitative studies) involving youth participation and decision-making in digital health interventions will be included.

### Exclusion Criteria

Studies primarily targeting adolescents and children, older adults, or those with wide age inclusion criteria (eg, 16-50 years) will be excluded in order to target results to YAs. Conference abstracts, commentaries, discussion papers, book editorials, guidelines, frameworks, and thesis papers are to be excluded. Relevant secondary research, including systematic reviews, scoping reviews, critical reviews, and meta-analyses, will be excluded but pooled for subsequent citation chaining.

### Gray Literature

The developed search strategy will be comprehensive to identify both published and unpublished evidence. Although excluded from this study, gray literature will be collated and assessed separately. Identified gray literature, including conference papers and organizational frameworks or guidance documents, provides a valuable overview of distinct co-design processes and offers insight into safe and effective implementation [12,13,27]. Although not always specific to digital interventions or young adults, these frameworks are useful in understanding the principles of participatory research and may be adapted to suit alternative population groups and tools.

### Data Charting Process

To extract data on study and intervention characteristics, an adapted version of the JBI results extraction instrument will be used [28]. Independent quality assurance of included studies will be undertaken using the JBI critical appraisal tools for qualitative studies, randomized controlled trials, and quasi-experimental research [29]. A narrative synthesis of final studies will allow for insights to be mapped by digital health tools, co-design, or similar methodologies and their effectiveness regarding acceptability, feasibility, and usability, influence on health behaviors; or both.

As per the JBI Reviewer’s Manual [20], key information to be collected from each paper will include:

- authors
- country of origin and year of publication
- study population and sample size
- intervention type
- purpose of study (aim)

By way of addressing the aforementioned research questions, intervention-specific data will be extracted on the use of co-design or similar theories and frameworks, description of digital health tools, health field and outcome, key findings, and limitations.

It will be assumed that the “purpose” or aim of studies may be characterized by:

- a description of the *development* of a digital health tool using co-design or participatory design;
- evaluation of the *acceptability, feasibility, or usability* of a co-designed digital health tool; or
- investigation and reporting of *direct or desired* health impacts of a co-designed digital health tool.

Author statements or discussions regarding the potential to build credibility, trust, collaboration, and advocacy online will be summarized, and we will provide a commentary on the use of developed interventions to support engagement, reach, segmentation, and accessibility for YAs.

### Critical Appraisal of Individual Sources of Evidence

An independent critical appraisal of the included sources of information will be conducted in order to assess reliability, relevance, and value [30]. The JBI’s critical appraisal tools for qualitative studies, randomized controlled trials, and quasi-experimental research will be used by 2 independent authors to guide the quality assurance process. The extent of co-design reporting will be added as an additional aspect of quality assurance, with those classified as “weak” to be reassessed for inclusion. Disagreements will be resolved by discussion with reviewers. Results will be compared and used in data synthesis to classify papers by quality.

### Data Synthesis

Due to the potentially large volume, breadth, and heterogeneity of material included in a scoping review, it is not possible to predetermine the optimal method of collating and reporting the results. The evidence may be presented in one or multiple of the following formats: narrative, table, or visual (eg, map or diagram). A draft table outlining key data extracted from each study (Tables 1 and 2) has been developed for reference [31].

**Table 1:** Exemplar tabular data representation (types of available evidence)^a^.

First author (Year)	Country sampling	Intervention (digital tool)	Purpose/aim	Key findings
Name (YYYY)	Country, population, sample size	App, website, online screening tool	Feasibility/acceptability, tool development, health impact	Engagement, health status, behavior, feasibility

^a^Adapted from Casu et al [31], which is published under Creative Commons Attribution 4.0 International License [32].

**Table 2:** Exemplar tabular data representation (cocreation process).

Study	Cocreation group	Sessions	Aim of session
First author (YYYY)	FU^a^ or HP^b^	Type (n)	For example, exploration of concept

^a^FU: future user.

^b^HP: health practitioner.

## Results

**Figure 1:**
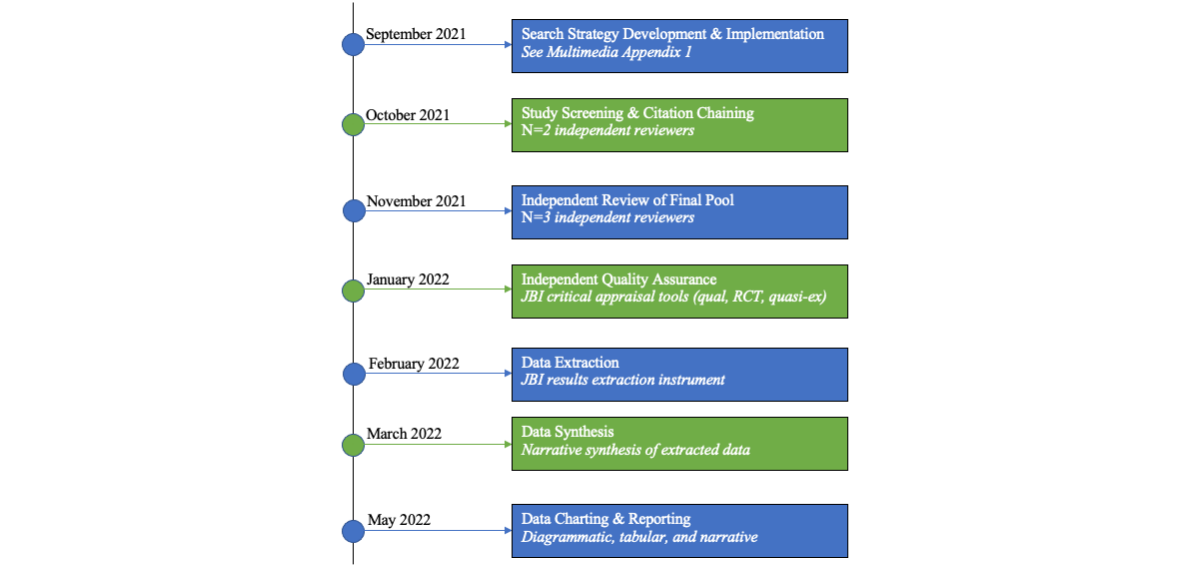
Timeline for conduct of scoping literature review. JBI: Joanna Briggs Institute; qual: qualitative; quasi-ex: quasi-experimental; RCT: randomized controlled trial.

Data collection commenced in October 2021, once the search strategies were developed. As of May 2022, we have identified 19 papers for analysis. Prior to conducting the narrative synthesis, for each study in the final pool, all relevant peer-reviewed primary papers associated with the digital tool of interest are being sought if available (eg, reports of co-design methodology vs intervention implementation). Additional studies identified will undergo data extraction and independent quality assurance. Once complete, the research team will embark on a narrative synthesis of the finalized pool. We expect results to be published by spring 2023 (Figure 1).

## Discussion

### Principal Findings

We hypothesize that a number of studies have implemented co-design or participatory design methodologies in distinct and creative ways to optimize engagement with and relevancy of developed digital tools with young adults. We foresee the exploration and dissemination of the insights regarding the strengths and challenges of participatory research with this age group, including effects on engagement, feasibility, and acceptability of developed digital tools. These insights will be summarized in order to inform the future cocreation of digital interventions for young people aged 15-35 years.

Prior participatory research indicates a number of key behaviors which have been targeted using co-designed digital tools [33-35]. As such, the types of behaviors likely to be targeted with the results from this review include risky health behaviors (eg, binge drinking, smoking), those associated with chronic disease states (eg, cancer, diabetes), and behaviors that have been identified as having positive influences on mental and physical well-being, such as healthy eating behaviors and physical activity.

### Comparison to Prior Work

As aforementioned, co-design research with a principal focus on the young adult population is scarce. Prior studies have examined the effect of inclusive research processes such as co-design on the engagement of children and adolescents with digital health interventions, and its potential as a mediator of positive health outcomes [10,16]. Similarly, we intend to explore recent participatory research involving an older age group in the development of digital health tools, as well as any associated influences on engagement and health outcomes.

### Strengths and Limitations

This protocol provides guidance for researchers who plan to conduct a similar style of investigation and promotes standardization of the scoping review process. Upon completion of data synthesis, the strengths and limitations of this review will be summarized.

### Future Directions

The results of this review will provide a synopsis of the breadth of literature on the employment of co-design methodology in the development of digital health interventions for young adults. We anticipate the provision of an overview of any gaps in this research area, as well as potential areas for further study. Regarding our plans for dissemination, we foresee the publication of the results of this review by spring 2023.

## Appendix

Multimedia Appendix 1 PRISMA-ScR (Preferred Reporting Items for Systematic Reviews and Meta-Analyses Extension for Scoping Reviews) checklist.

## Abbreviations

FU: future user
JBI: Joanna Briggs Institute
PCC: participants, concept, context
PRISMA-ScR: Preferred Reporting Items for Systematic Reviews and Meta-Analyses Extension for Scoping Reviews
ScR: scoping review
YA: young adult
